# Internet use and self-rated health among Chinese older adults: the role of social engagement and social support

**DOI:** 10.3389/fpubh.2024.1504940

**Published:** 2025-02-05

**Authors:** Pei-Qi Li, Guo-Yuan Sun, Xue-Xue Zhao, Zheng-Xin Hu, Kai-Peng Gan

**Affiliations:** ^1^School of Law and Political Science, Yunnan University of Finance and Economics, Kunming, China; ^2^Yunnan Key Laboratory of Service Computing, Yunnan University of Finance and Economics, Kunming, China

**Keywords:** internet use, self-rated health, social engagement, social support, older adults

## Abstract

This study examines how social engagement and social support affect association between internet use and older adults' self-rated health in Kunming. An analysis of 518 community-dwelling older adults reveals that internet use significantly enhances their self-rated health. Social engagement mediates this relationship, while social support strengthens the beneficial impact of social engagement on self-rated health. Additionally, social support amplifies the mediating role of social engagement, suggesting that greater social support enhances the benefits of social engagement on health outcomes. Our findings emphasize the significance of fostering social engagement and providing robust social support to improve older adults' health.

## 1 Introduction

In 1994, China officially embraced the internet. As the country's science and technology advanced rapidly, the internet era quickly emerged, attracting an increasing number of users. According to a study by Yang et al., by June 2020, the internet user base in China had grown to 940 million, representing a penetration rate of 67.0% (1). Over the same time frame, the percentage of older persons utilizing the internet also rose, with those aged 60 and beyond rising from 6.7% in March to 10.3% in June. In China, 60.61% of adults and 64.69% of minors possess at least basic digital literacy and skills, while 16.26% of adults and 12.55% of minors have advanced digital literacy and skills (75). In an era of accelerating social digitalization, digital literacy is increasingly regarded as a prerequisite for meaningful participation in various aspects of modern society, including daily life, education, and work (2). The widespread use and adoption of the internet have greatly enriched people's lives, becoming a tool for more and more older adults to obtain news and communicate with relatives and friends (3), integrating into their daily lives. Many studies have shown that internet use significantly affects older adults' life satisfaction, self-rated health, and subjective wellbeing (4, 5, 77, 82). The literature notes that high technology has two sides, meaning the internet can both promote health and have negative effects (76). Older adults who use the internet tend to report better health, a reduced risk of chronic diseases, and an inverse association with depression, contributing positively to their mental health (6–9). However, some scholars have suggested that internet use may have a notably negative effect on older adults' health (10, 11). Consequently, the impact of internet use on older adults' health remains a topic of debate.

Additionally, many scholars have examined the role of intermediate variables such as family support, support from friends, social engagement, social contact, and interpersonal relationships in the association between internet use and older adults' health (12, 13, 83). Internet use allows older adults to participate in society without being affected by geographical restrictions, and those who use the internet more frequently experience lower levels of mental distress than those who use it infrequently or not at all (14). The internet allows older adults to maintain contact with relatives and friends who are geographically distant, fostering new connections with the broader world. This not only reinforces their ties to external social networks but also enhances communication with loved ones, enabling them to receive more social support, information, and assistance from well-acquainted family and friends. Enhanced perception of social support among older persons is positively associated with increased resilience, thereby leading to improved health outcomes (78). Thus, the linkage between internet use and older adults' health may be greatly influenced by social engagement and social support. Therefore, the internet use will significantly affect the health of older adult individuals, yet the mechanisms by which it impacts their health require further exploration.

Based on the above issues and discussions, the present study investigates how internet use affects older adults in China, exploring the mediating roles of social participation and social support in the connection between internet use and self-reported health. The objectives and contributions of our study are as follows. First, this study provides new evidence for the theory of health promotion by examining the connection between internet usage and self-reported health among older adult individuals in China, further supporting the view that internet use contributes to health promotion. Second, the present study incorporates social participation into the analytical framework and examines its role as a mediator in the link between internet use and self-reported health, thus deepening our understanding of the mechanisms linking internet use to older adults' self-rated health. By assessing the mediating impact of social participation, this study explains why older adults who frequently use the internet tend to be healthier, thereby validating the positive role of social participation theory in promoting the health of older adults. Finally, drawing on social capital theory, this study explores the indirect moderating role of social support in the relationship between internet use and self-reported health. It broadens the scope of how internet use affects the self-rated health of older adults and contributes to the literature on social support theory within the context of older adult health.

## 2 Literature review and research hypotheses

### 2.1 Internet use and self-rated health of older adults

Digital literacy is a broad concept that encompasses digital cognition, digital thinking, and digital technology, and is significantly related to the subjective health status of older adults (15). Digital literacy is also characterized as the capability to navigate the internet and smartphones to search for, assess, utilize, share, and generate content using digital technologies (16). As the global population ages, leveraging the internet and digital technology to enhance older adults' health has become a crucial strategy for promoting healthy aging (17). Internet-based weight loss programs have demonstrated clinically significant weight reduction, marked improvements in depressive symptoms, and a lower risk of developing clinical depression (18). Furthermore, using the internet to influence lifestyle factors like diet, sleep, exercise, smoking, and alcohol consumption among older Chinese adults has been shown to reduce depression and enhance overall health (19). These studies suggest that older adults' health will be positively affected by internet use.

Digital and e-health literacy empowers individuals to efficiently seek information while adopting a critical and open-minded approach, and to apply newly gained knowledge to enhance their mental wellbeing (20). Previous research demonstrates that internet use can benefit older adults' health to some extent. One important theory in this regard is the health promotion theory, which posits that individuals who use the internet tend to search for health-related information online more frequently, thereby promoting their own health status (21). For instance, several studies indicate that active internet usage has a beneficial influence on the mental wellbeing, management of chronic illnesses, and levels of depression in older adults (22, 23). Internet use not only enhances mental wellbeing but also contributes to physical health improvements (24, 25). For example, Research conducted by Guo et al. revealed a significant reduction in the occurrence of depression among older adult individuals who used the internet, resulting in enhancements to their mental wellbeing. Based on these findings, we expect that older adults' wellbeing and health will be significantly affected by internet use (79, 80). Consequently, the following hypothesis is proposed.

**Hypothesis 1:** Internet use significantly benefits older adults' self-rated health.

### 2.2 The mediating role of social engagement

The term “social engagement” does not have a standardized definition. Typically, social engagement is characterized by participation in activities that involve interacting with others within a society or community (26). Levasseur et al. analyzed 43 articles using keywords related to social engagement, defining it as activities in which individuals engage with others in their society (27). According to the main objectives of these social activities, six levels of personal involvement with others have been delineated, from close to more distant: (1) participating in activities with the goal of connecting with others, (2) spending time with others, (3) interacting with others without specific activities, (4) engaging in shared activities with others, (5) assisting others, and (6) contributing to society. The Digital Literacy Task Force of ALA defines digital literacy as “the ability to use information and communication technologies to find, evaluate, create, and communicate information, requiring both cognitive and technical skills.” Digital literacy also includes understanding digital tools and using them through social participation for communication and collaboration (28). Therefore, we redefine “social participation” as an integrated set of behaviors in which individuals engage in activities or interact with others in society or the community, encompassing “digital literacy” and “digital skills.”

As a key factor for health, digital literacy is emphasized for its close interaction with other intermediate health factors (29). The linkage between internet use and health can be understood through social engagement theory, which highlights the significance of interpersonal relationships, social interactions, and participation in activities within a societal framework (30, 31). According to this theory, active participation in social events offers older adult individuals both financial and emotional assistance, as well as a feeling of self-identity and self-worth through interpersonal connection and interaction. This, in turn, contributes to enhanced mental health (32).

When considering the topic of digital literacy, it naturally refers first to the more social aspects of community life, such as connecting with others, communicating, and interacting with others through digital tools (33). The use of information and communication technologies is positively correlated with healthy aging (34). As internet use becomes increasingly integral to daily social activities such as managing living expenses, online shopping, and accessing financial services, thus, its role in promoting middle-aged and older adults' social engagement and healthcare behavior is becoming increasingly important (35). Older adults must establish new social connections to adapt to the loss of traditional social roles and interactions (36). Furthermore, active social engagement helps sustain vitality and motivation across physical, psychological, and social dimensions (37). Social engagement, therefore, acts as a vital connection between internet use and health outcomes in this demographic. The internet facilitates continuous and convenient contact with relatives and friends, overcoming barriers of time and space, thereby enhancing social communication and interaction, which is essential for maintaining physical and mental health. Additionally, internet technology mitigates information asymmetry, enabling older adults to access and understand social information more effectively, thereby enhancing their social interaction and enhancing their general wellbeing (38). The relationship between internet use and health status among older persons is mediated by social engagement (39). Working from this underlying assumption, we put up the subsequent hypothesis.

**Hypothesis 2:** Social interaction plays a mediating role in the association between internet usage and the health of older persons.

### 2.3 The moderating role of social support

Essentially, social support refers to the assistance obtained through interpersonal relationships (40). Scholars have described it in various ways. Veiel defines social support as “any verbal or non-verbal information or material help provided by groups or individuals, which leads to positive emotional effects or behaviors” (41). Miao et al. describe social support as the emotional and material support individuals receive from others when facing stress or challenges, creating a sense of being cared for and valued during interpersonal interactions (42). Carri et al. define social support as specific and genuine emotional or informational positive social interactions available to individuals (43). Such robust support from family, friends, and others can improve both physical and mental health (44).

Digital literacy is associated with improvements in wellbeing, social support, and quality of life (45). Social capital, encompassing social support and reciprocity, is positively correlated with health outcomes. This suggests that older adults who receive more social support (including moral and material assistance) and exhibit greater reciprocity (willingness to reciprocate with relatives, friends, neighbors, and strangers) are likely to experience better health (46). Digital literacy empowers older adults to avoid isolation, sustain social connections, and engage in a wide range of activities (47). The use of information and communication technologies by older adults is associated with fewer symptoms of depression, higher self-rated health, and greater subjective wellbeing (48). The village model represents an emerging, consumer-driven approach to social support, aimed at enhancing social participation, independence, and wellbeing among older community members through a combination of social activities, volunteer opportunities, service referrals, and direct assistance (49). Increasing social support can encourage older adults to engage in a variety of community activities, which is beneficial for healthy aging (50).

Activity theory posits that while older adults may not be in the same mental state as younger individuals, they can still enhance their self-worth and achieve a sense of social identity through active participation in social activities (51). In urban settings, social networks and engagement are related to positive self-rated health (52). Previous research indicates that greater social support leads to higher levels of older adults' social engagement. Effective and timely social support promotes greater social engagement, which is positively correlated with improved health. Consequently, a high level of social support is correlated with greater social engagement and improved health outcomes for older individuals. Building on this understanding, we put forward the following hypothesis.

**Hypothesis 3:** Relationship between social engagement and self-rated health of older individuals is moderated by social support.

Based on Hypotheses 2 and 3, we expect that the effects of internet use on the self-rated health of older individuals may be moderated by social support (SSM). More precisely, elevated degrees of social support can enhance the influence of social engagement in moderating the connection between internet usage and health results for adults of advanced age. When older adults receive substantial support from family, friends, and relatives, they are likely to be more engaged in health-promoting social activities. Conversely, a lack of social support may result in reduced social engagement and potentially poorer health outcomes. Accordingly, the present study aims to examine the impact of different levels of social support on the mediating function of social engagement in the correlation between internet usage and self-assessed health. Thus, the presented hypothesis is as follows.

**Hypothesis 4:** Social support will moderate the indirect of internet use on older adults' self-rated health through social engagement.

## 3 Methods

### 3.1 Sample and participants

To select communities and older adult groups closely related to the present study, we employed purposive sampling to ensure that the research sample adequately covers older adult individuals from different types of communities. From September 10 to November 26, we selected five main districts from seven districts in Kunming City. Then, for each district, we randomly selected a community with more than 40 buildings as the survey unit. The five communities selected for this survey are all residential communities built after 2010, which include residents from various professions and social classes. Before commencing the survey, the researchers recruited six university teachers and 10 students, providing them with prior training on the survey methods. The researchers then reached out to relevant officials in five communities, informing them of the research objectives and data collection methods, and invited them to act as contacts to assist in recruiting older adult participants for the survey. Subsequently, the researchers ensured that all older adult participants voluntarily agreed to participate and conducted the data collection through one-on-one interviews. In the selected survey communities, we used a simple random sampling method for data collection. Out of 600 distributed questionnaires, 558 were returned. After excluding incomplete and invalid responses, the final sample comprised 518 valid questionnaires, resulting in a valid recovery rate of 86%. The study included 50.4% males, 49.6% females, and 66.6% individuals who had achieved a bachelor's degree or above. Additionally, 90% reported using the internet for more than 2 h per day.

### 3.2 Measures

#### 3.2.1 Internet use

Internet use was evaluated using a five-item scale created by Ma (53). Items included “downloading videos, music, or software” and “searching for information.” The Cronbach's alpha for this scale was 0.936 in the current study.

#### 3.2.2 Social engagement

We measured social engagement using an eight-item scale developed by Kim and Park (81). Examples of items are “How often do you meet with friends, relatives, or neighbors who live nearby?” and “How often do you participate in leisure, cultural, or sports activities?” The Cronbach's alpha for this scale was 0.939.

#### 3.2.3 Social support

Social support was assessed using a ten-item scale created by Xiao (54). Sample items include “When you encounter emergency situations, what sources of comfort and concern have you received?” and “How do you seek help when you are in trouble?” The Cronbach's alpha for this scale was 0.986.

#### 3.2.4 Self-rated health

We assessed self-rated health using a five-item scale created by Lee et al. (55), which was used and validated by some previous research (56, 57). Two examples of sample items are “I have experienced a positive and upbeat mood” and “I have felt physically active and energetic.” The study reported a Cronbach's alpha coefficient of 0.964 for the scale.

### 3.3 Analysis strategy

Adhering to the suggestions of Tang and Li (58), we implemented a two-step analytical approach. First, the variables were evaluated for discriminant validity using confirmatory factor analysis with AMOS. Subsequently, dependence and correlation analyses were conducted with SPSS (version 26.0) to investigate the direct impact of internet usage on the self-assessed health of older adult individuals. The present study examined the mediating role of social engagement and the moderating role of social support using the SPSS PROCESS macro version 4.1. The findings were visually represented through effect diagrams.

## 4 Results

### 4.1 Confirmatory factor analysis

An assessment was conducted to determine the discriminant validity of the main variables by comparing five different models with the four-factor benchmark model (Table 1). The best fit indices (χ^2^/df = 3.469, RMSEA = 0.069, CFI = 0.962, TLI = 0.953, NFI = 0.948) of the four-factor model, as shown in Table 2, demonstrate its superior performance compared to the other models. The findings indicate that the four-factor model demonstrates a better data fit than other models. The two-factor model displayed a less satisfactory fit to the data, with coefficients of determination (χ^2^/df = 10.680, RMSEA = 0.137, CFI = 0.851, TLI = 0.817, NFI = 0.838).

**Table 1:** Confirmatory factor analysis: discriminative validity.

**Models**	**χ^2^/df**	**RMSE**	**CFI**	**TLI**	**NFI**
Four-factor model	3.469	0.069	0.962	0.953	0.948
**Three-factor model**					
Model a: IU+SE, SS, SH	4.637	0.084	0.944	0.931	0.930
Model b: IU, SE + SS, SH	6.027	0.099	0.923	0.905	0.909
Model c: IU, SE, SS + SH	9.730	0.109	0.906	0.884	0.892
Two-factor model: IU+SE, SS+SH	10.680	0.137	0.851	0.817	0.838
One-factor model: IU+SE+SS+SH	13.866	0.158	0.801	0.757	0.790

IU, Internet Use; SH, Self-rated Health; SE, Social Engagement; SS, Social Support.

+Means that two factors merge into one variable.

**Table 2:** Means, standard deviations, and correlations among the study variables.

**Variables**	**1**	**2**	**3**	**4**	**5**	**6**	**7**
1. Gender	1						
2. Age	0.007	1					
3. Education	−0.001	−0.212^**^	1				
4. Internet use	−0.020	−0.007	0.026	1			
5. Social engagement	−0.012	0.080	−0.137^**^	0.713^**^	1		
6. Social support	−0.010	0.013	−0.096^*^	0.676^**^	0.700^**^	1	
7. Self–rated health	−0.023	0.188^**^	−0.163^**^	0.530^**^	0.592^**^	0.624^**^	1
Mean	0.50	3.21	2.58	3.916	3.647	3.403	3.475
SD	0.500	0.900	0.835	0.8236	0.850	0.975	0.980

N = 552.

^*^p < 0.05.

^**^p < 0.01.

### 4.2 Descriptive statistics and correlation

The correlation analysis of the variables is displayed in the Pearson correlation coefficient matrix (Table 2). The results indicate significant correlations between the control variable, education level, and social engagement, social support, and self-rated health. Specifically, internet use significantly and positively affected self-rated health (*r* = 0.530, *p* < 0.01), social engagement (*r* = 0.713, *p* < 0.01), and social support (*r* = 0.676, *p* < 0.01), supporting Hypothesis 1. Furthermore, self-rated health was positively related to social engagement (*r* = 0.592, *p* < 0.01) and social support (*r* = 0.624, *p* < 0.01). Social engagement also demonstrated a positive correlation with social support (*r* = 0.700, *p* < 0.01). These results indicate that the moderating effect of social support deserves further investigation.

### 4.3 Hypothesis testing

The result in Table 3 demonstrates that internet usage greatly improves the self-assessed health of older adult individuals (*b* = 0.635, *p* < 0.001). Thus, Hypothesis 1 was supported. Additionally, the results show a strong positive correlation between internet usage and social engagement, which acts as a mediator (*b* = 0.740, *p* < 0.001), as well as between social engagement and self-assessed health (*b* = 0.440, *p* < 0.001). The indirect impact of internet use on self-rated health through social engagement is significant [B = 0.326, 95% confidence interval [CI]: [0.237–0.418]], supporting Hypothesis 2 and suggesting that social engagement partially mediates the effect of internet use on self-rated health.

**Table 3:** Results for mediator effect test.

	**Social engagement**	**Self–rated health**	
**Variable**	**Mode l**	**Mode 2**	**Mode 3**
Constant	0.962 (0.185)^***^	0.870 (0.254)^***^	0.447 (0.247)
Gender	0.003 (0.051)	−0.027 (0.070)	−0.0281 (0.067)
Age	0.051 (0.029)	0.175 (0.040)^***^	0.153 (0.038)^***^
Education	−0.147 (0.031)^***^	−0.167 (0.043)^***^	−0.102 (0.042)^*^
Internet use	0.740 (0.031)^***^	0.635 (0.043)^***^	0.309 (0.059)^***^
Social engagement			0.440 (0.058)^***^
Total effect		0.635 [0.551, 0.719]	
Direct effect		0.309 [0.194, 0.425]	
Indirect effect		0.326 [0.237, 0.418]	
*R* ^2^	0.535^***^	0.337^***^	0.405^***^

Bootstrap size = 5,000.

CI, confidence interval.

^*^p < 0.05.

^***^p < 0.001.

Table 4 demonstrates that older adults' self-rated health is positively affected by social engagement and social support (*b* = 0.263, *p* < 0.001; *b* = 0.378, *p* < 0.001). Furthermore, the interaction between social engagement and social support significantly affects self-rated health (*b* = 0.094, *p* < 0.01), supporting Hypothesis 3. These findings indicate that the influence of internet use on self-rated health is influenced by social support. Specifically, greater levels of social support enhance the beneficial effects of social engagement on self-rated health.

**Table 4:** Results for moderating effect test.

**Variable**	***B***	**SE**	***P***	**Boot LLCI**	**Boot ULCI**
**Outcome variables: self-rated health**					
Constant	2.535	0.278	0.000	1.988	3.082
Gender	−0.031	0.062	0.616	−0.154	0.091
Age	0.160	0.036	0.000	0.090	0.231
Education	−0.071	0.039	0.073	−0.148	0.007
Internet use (X)	0.145	0.059	0.014	0.029	0.261
Social engagement (M)	0.263	0.059	0.000	0.147	0.379
Social support (W)	0.378	0.048	0.000	0.284	0.473
M × W	0.094	0.031	0.002	0.034	0.155

R^2^ = 0.480, F-value = 67.219.

Bootstrap size = 5,000.

Boot SE, bootstrapping standardized error; LL, low limit; UL, upper limit; CI, confidence interval.

p < 0.05.

p < 0.01.

p < 0.001.

To further investigate the moderating role of social support, we analyzed variations in social support by adding or subtracting one standard deviation. Table 5 shows that the 95% confidence interval for the moderated effect is [0.150, 0.373], excluding zero. Therefore, the presence of social support serves to reduce the impact of social engagement on the correlation between internet usage and self-assessed health, so confirming Hypothesis 4.

**Table 5:** Results for the index of moderated mediation and conditional indirect effects.

**Moderator: social support**	**Effect/ index**	**Boot SE**	**Boot LLCI**	**Boot ULCI**
**Conditional indirect effects [95% CI]**				
M–SD (−0.975)	0.126	0.058	0.011	0.239
Mean	0.195	0.051	0.093	0.292
M+SD (+0.975)	0.263	0.056	0.150	0.373
**Index of moderated mediation**				
Social support	0.070	0.026	0.018	0.122

Bootstrap size = 5,000.

Boot SE, bootstrapping standardized error; LL, low limit; UL, upper limit; CI, confidence interval.

p < 0.05.

p < 0.01.

p < 0.001.

**Figure 1:**
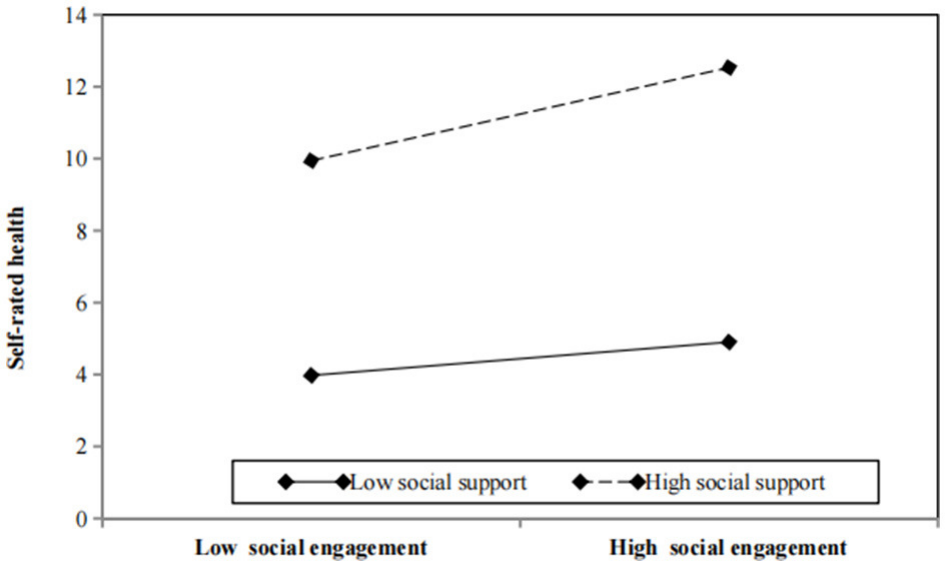
Interaction plot of social engagement and social support predicting self-rated health.

Furthermore, Figure 1 illustrates the moderating impact of different degrees of social support. The graph illustrates that the gradient for high social support is more pronounced compared to that for low social support, indicating that improved health outcomes and more social engagement among older persons are related to higher levels of social support. The results indicate that a strong level of social support amplifies the immediate influence of social engagement on self-assessed health, whereas a somewhat smaller impact is observed with poor social support. Therefore, it can be proposed that social support plays a beneficial role in moderating the influence of social engagement as a mediator in the correlation between internet usage and the self-assessed health of older individuals. This implies that a greater level of social support is associated with more social engagement and improved health results.

## 5 Discussion and conclusions

The present study investigates the correlation between internet usage and the self-assessed health status of older adult individuals, as well as the underlying mechanisms that contribute to this association. Firstly, the study reveals a significant positive relationship between internet use and older adults' self-rated health. Specifically, greater internet use is associated with better health outcomes within this demographic. This finding aligns with previous research suggesting that internet use can enhance access to health information for older adults, facilitate social interaction, encourage physical exercise, and improve social networks and planned behaviors, which are key pathways through which digital literacy enhances health, ultimately promoting better health (59–61, 79, 80). Consequently, our study contributes additional empirical evidence supporting the beneficial influence of internet use on older adults' self-rated health.

Furthermore, the study establishes social engagement as a mediating element in the correlation between internet usage and the self-assessed health of older individuals. This finding supports previous studies (62, 63), which established the role of social engagement in mediating the aforementioned link. Usage of the internet improves the cognitive processing of information and decreases doubt regarding online resources. Furthermore, fostering a supportive social and familial environment for older adults' internet usage will increase their social engagement, mitigate feelings of loneliness and aging-related stress, and improve their overall self-rated health (64, 84). Thus, the present study provides more support for the mediatory function of social interaction in the association between internet usage and self-rated health in the older adult population.

Finally, the study demonstrates that social support acts as a moderator in the connection between social engagement and the self-assessed health of older adult individuals. More precisely, older adult individuals who beget substantial social support are more inclined to engage in social activities, which in turn result in enhanced self-assessed health. The present study is the first to confirm the moderating effects of social support in this dynamic, underscoring how it amplifies the positive impact of social engagement on self-rated health (65, 66).

### 5.1 Theoretical implications

The present study offers some theoretical implications. In the first instance, it enhances the existing body of knowledge on the correlation between internet use and the self-assessed health of older adult individuals, therefore offering valuable understanding of the causal relationship between these factors. Despite ongoing debate about this causal relationship (67, 68), our research, based on a sample from urban areas in southwest China, expands understanding and offers a foundation for future studies.

Secondly, the study elucidates the intermediary function of social engagement in the correlation between internet use and the self-assessed health of older adult individuals. Few empirical research has examined this mechanism, and while some research has explored other mediators (69, 70), the role of social engagement has not been extensively investigated. This study addresses this gap by identifying social engagement as a key mediator.

Furthermore, this study emphasizes the beneficial influence of social support on the self-assessed health of older adult individuals who utilize the internet, therefore expanding the scope of social support theory. Existing literature has predominantly focused on social support as a mediating factor in studies related to internet usage (71–73). This study aims to explore the significance of social support in moderating the relationship between internet use and self-rated health. Additionally, it seeks to examine how social support influences the mediation impact of social engagement.

### 5.2 Practical implications

Our study also has several practical implications for policy-makers. First, given the significant impact of internet use on self-rated health among Chinese older adults, it is recommended that internet access and training be prioritized. Cultivating digital literacy and digital skills among older adults, strengthening the infrastructure and training for digital technologies such as the internet, and increasing their access to online health resources can enhance their mental wellbeing and integrate internet use with physical activities that benefit their overall health.

Second, addressing the social isolation experienced by older adults due to physical limitations and reduced interpersonal connections is crucial. Internet-based communication tools and entertainment apps can help bridge this gap, fostering greater social engagement and access to information. Managers should promote both online and offline social activities to alleviate negative emotions and improve health outcomes.

Ultimately, social support plays a vital role in enhancing the connection between social engagement and self-assessed health in an older population. Policymakers should optimize institutional support in areas such as health services and pensions, and encourage additional forms of social support from community members, friends, and relatives to complement governmental efforts (74, 78).

### 5.3 Limitations and future research

Our research has several limitations. First, the data is based on a questionnaire survey from an urban community in southwest China, which may not fully represent the situation in other regions or rural areas. The socio-economic and cultural differences between southwest China and other regions, as well as between urban and rural communities, may yield different results. Furthermore, although this study focuses on the functions of social engagement and social support, it does not investigate other possible factors that could impact the correlation between internet use and self-assessed health measures. Future research should investigate these additional factors and consider diverse geographic and demographic contexts.

Conclusively, this study provides significant findings on the beneficial impact of internet use on the self-assessed health of older adult individuals, emphasizing the intermediary function of social engagement and the moderating influence of social support. The results of this study add to the current body of knowledge and offer practical suggestions for improving the self-assessed health of older adult individuals by means of enhanced internet connectivity and reinforced social support networks. To further the understanding of the association between internet use and self-rated health among older persons, future research should investigate additional variables and situations, building upon current findings.

## Data Availability

The original contributions presented in the study are included in the article/Supplementary material, further inquiries can be directed to the corresponding author.
