# Supplementary material for: Internet use and self-rated health among Chinese older adults: the role of social engagement and social support
Source: Front Public Health. 2025 Feb 5;12:1504940. doi: 10.3389/fpubh.2024.1504940 (PMC11835876; doi:10.3389/fpubh.2024.1504940)
Supplement: Supplementary file 1 [file Data_Sheet_1.docx]

Questionnaire number： ⬜⬜⬜

**Questionnaire on the living conditions of the elderly in the community in Kunming**

Hello! This volume is designed to solve the basic living conditions of the elderly in the community, and we sincerely ask for your support and cooperation. In this survey, I pledge that any information you provide will be used for research purposes as a whole, not for commercial or other political purposes, without privacy, and without distinction between right and wrong. Thank you!

Please read this questionnaire carefully and put a "√" on the item number you recognize.

**1. Basic information (please tick "√" on the eligible options)**

A. What is your gender?

(1) Male (2) Female

B. What is your age?

(1) 50-60 years old (2) 61-70 years old (3) 71-80 years old

(4) 81-90 years old (5) 91 years old or older

C. What is your level of education?

(1) High school and below, (2) junior college, (3) bachelor's degree, (4) master's degree or above

D. Do you use internet time?

(1) Less than 2 hours (2) 2-5 hours (3) 5-8 hours (4) 8-12 hours (5) More than 12 hours

E. What is your socioeconomic status?

(1) Lower (2) Lower middle (3) Middle (4) Upper middle (5) Upper

F. What is your annual income? Yuan (RMB), divided into grades

(1) Less than 25,000 yuan, (2) 25000-35000 (3)35000-50000, (4)50000-70000, (5)70000-90000, (6) 90000-110000, (7)110000-130000, (8)130000-150000,

(9)150000-180000, (10)180000-200000, (11)200000-250000, (12) more than 250,000 yuan

**2. The following is your basic opinion about your lifestyle; please choose the answer according to your real thoughts.**

| **Please read each sentence carefully to see if it suits your situation, and put "√" on the corresponding number.** | **Very much not** | **Not true** | **Neither agree nor disagree** | **true** | **Very much in line with** |
| --- | --- | --- | --- | --- | --- |
| **Social support** |  |  |  |  |  |
| 16. You can get support and help from close friends. | **1** | **2** | **3** | **4** | **5** |
| 17. You have lived with family, classmates, colleagues, or friends for the past year. | **1** | **2** | **3** | **4** | **5** |
| 18. Your neighbors care about you. | **1** | **2** | **3** | **4** | **5** |
| 19. Your current or former co-workers care about you. | **1** | **2** | **3** | **4** | **5** |
| 20. You can get support and care from family members (couples or lovers, parents, daughters, siblings, etc.). | **1** | **2** | **3** | **4** | **5** |
| 21. In the past, when you were in a difficult situation, you have received financial support and help from others (family, relatives, colleagues, etc.) to solve practical problems. | **1** | **2** | **3** | **4** | **5** |
| 22. In the past, when you were in a difficult situation, you used to receive comfort and care from others (family, relatives, co-workers, etc.). | **1** | **2** | **3** | **4** | **5** |
| 23. When you have a problem, you can confide in someone else (a friend or someone close to you). | **1** | **2** | **3** | **4** | **5** |
| 24. When you are troubled, you can turn to others (friends or close friends) for help. | **1** | **2** | **3** | **4** | **5** |
| 25. You often participate in the activities of social groups (such as party organizations, religious organizations, trade unions, student unions, etc.). | **1** | **2** | **3** | **4** | **5** |
|  |  |  |  |  |  |
| **Please read each sentence carefully to see if it suits your situation, and put "√" on the corresponding number.**  **"1" means the sentence "almost nothing"; "2" means "less"; "3" means "general"; "4" means "more frequent"; "5" means "very frequent".** | **almost nothing** | **less** | **general** | **more frequent** | **very frequent** |
| **Social engagement** |  |  |  |  |  |
| 1. How often do you meet up with friends, relatives, or neighbors who live nearby? | **1** | **2** | **3** | **4** | **5** |
| 2. What types of organizations are you currently involved in? religion | **1** | **2** | **3** | **4** | **5** |
| 3. Socializing (senior clubs, rotating savings and credit associations, etc.); | **1** | **2** | **3** | **4** | **5** |
| 4. Leisure/Culture/Sports; | **1** | **2** | **3** | **4** | **5** |
| 5. Gatherings; | **1** | **2** | **3** | **4** | **5** |
| 6. Volunteering; | **1** | **2** | **3** | **4** | **5** |
| 7. Political parties/civic organizations/interest groups; | **1** | **2** | **3** | **4** | **5** |
| 8. Others (e.g. watching TV, etc.). | **1** | **2** | **3** | **4** | **5** |
| **Internet use** |  |  |  |  |  |
| 1.downloading videos, music or software; | **1** | **2** | **3** | **4** | **5** |
| 2.urfing websites such as YouTube and online shopping for leisure; | **1** | **2** | **3** | **4** | **5** |
| 3.playing online games | **1** | **2** | **3** | **4** | **5** |
| 4.searching for information | **1** | **2** | **3** | **4** | **5** |
| 5.social networking (visiting social platforms such as Facebook, Twitter and Weibo) | **1** | **2** | **3** | **4** | **5** |
| 6.sending instant messages/emails; | **1** | **2** | **3** | **4** | **5** |
| 7.visiting forums/discussion groups | **1** | **2** | **3** | **4** | **5** |

**3. The following is your emotional state in the past year; please choose the answer according to your true thoughts.**

| Please read each sentence carefully to see if it suits your situation, and put "√" on the corresponding number.  "1" means the phrase "never"; "2" means "occasionally"; "3" means "sometimes"; "4" means "often"; "5" means "always". | never | occasionally | sometimes | often | always |
| --- | --- | --- | --- | --- | --- |
| **Please read each sentence carefully to see if it suits your situation, and put "√" on the corresponding number.** | **Very much not** | **Not true** | **Neither agree nor disagree** | **true** | **Very much in line with** |
| **Self-rated health** |  |  |  |  |  |
| D1. Feeling nervous | **1** | **2** | **3** | **4** | **5** |
| D2. Feeling easily distressed or angry | **1** | **2** | **3** | **4** | **5** |
| D3. Feeling depressed and depressed | **1** | **2** | **3** | **4** | **5** |
| D4. I feel inferior to others | **1** | **2** | **3** | **4** | **5** |
| D5. Difficulty sleeping, such as difficulty falling asleep, waking up easily or waking up early | **1** | **2** | **3** | **4** | **5** |

Thank you again for your support and help!
